# Electronic structure and core electron fingerprints of caesium-based multi-alkali antimonides for ultra-bright electron sources

**DOI:** 10.1038/s41598-019-54419-0

**Published:** 2019-12-04

**Authors:** Caterina Cocchi, Sonal Mistry, Martin Schmeißer, Raymond Amador, Julius Kühn, Thorsten Kamps

**Affiliations:** 1 Humboldt-Universität zu Berlin, Physics Department, 12489 Berlin, Germany; 2 Humboldt-Universität zu Berlin, IRIS Adlershof, 12489 Berlin, Germany; 3 Helmholtz-Zentrum Berlin, 12489 Berlin, Germany

**Keywords:** Condensed-matter physics, Theory and computation, Electronic properties and materials, Characterization and analytical techniques

## Abstract

The development of novel photocathode materials for ultra-bright electron sources demands efficient and cost-effective strategies that provide insight and understanding of the intrinsic material properties given the constraints of growth and operational conditions. To address this question, we propose a viable way to establish correlations between calculated and measured data on core electronic states of Cs-K-Sb materials. To do so, we combine first-principles calculations based on all-electron density-functional theory on the three alkali antimonides Cs_3_Sb, Cs_2_KSb, and CsK_2_Sb with x-ray photoemission spectroscopy (XPS) on Cs-K-Sb photocathode samples. Within the *GW* approximation of many-body perturbation theory, we obtain quantitative predictions of the band gaps of these materials, which range from 0.57 eV in Cs_2_KSb to 1.62 eV in CsK_2_Sb and manifest direct or indirect character depending on the relative potassium content. Our theoretical electronic-structure analysis also reveals that the core states of these systems have binding energies that depend only on the atomic species and their crystallographic sites, with largest shifts of the order of 2 eV and 0.5 eV associated to K 2*p* and Sb 3*d* states, respectively. This information can be correlated to the maxima in the XPS survey spectra, where such peaks are clearly visible. In this way, core-level shifts can be used as fingerprints to identify specific compositions of Cs-K-Sb materials and their relation with the measured values of quantum efficiency. Our results represent the first step towards establishing a robust connection between the experimental preparation and characterization of photocathodes, the *ab initio* prediction of their electronic structure, and the modeling of emission and beam formation processes.

## Introduction

The future of bright electron sources relies on the close interplay between theoretical understanding and experimental verification of the material characteristics and their role for the electron beam properties. Photoemission based electron sources are the enabling concept for many electron accelerator based applications such as the free-electron laser or ultra-fast scattering instruments^1^. Furthermore, they are essential components for a plethora of technological devices ranging from photovoltaic cells to radiation detectors. For accelerator applications great progress has been achieved in recent years with regards to the preparation and characterisation of photoemission electron sources, especially given the boundary conditions present in the harsh environment of the particle accelerator. The electron source, *i.e*., the photocathode, shares the vacuum environment of the first accelerating section and is subject to strong radio-frequency or static electric fields. In parallel with the experimental progress in the preparation of photocathodes it is also now possible to understand many aspects of the process of the generation of the electron pulses after emission. What is still missing is a deeper understanding of the intrinsic material properties and their role in the formation of the initial electron beam characteristics. There is also lack in knowledge about the effects of the growth process or contamination typically present in the accelerator environment. Right now the frontier of materials for accelerator applications is represented by semiconductors with maximised quantum efficiency (QE) in the visible region and minimised transverse energy (MTE)^2^. Compared to simple metals or conventional semiconductors such as GaAs, they feature relatively low electron affinity and band gap, both on the order of 1 eV, which are key requirements for high QE in the visible band. Furthermore, their vacuum requirements are more modest compared with GaAs photocathodes^1^. Motivated by these intriguing properties, a number of groups worldwide are striving to produce and operate photocathodes for particle accelerators based on multi-alkali antimonides^3–8^.

For example, at the Helmholtz-Zentrum Berlin (HZB), Cs-K-Sb photocathodes have been grown, characterised, and transferred into the photoelectron gun of the energy-recovery linac (ERL) bERLinPro. In the photoelectron gun the photocathode is placed inside a superconducting radio-frequency (SRF) cavity at the location of peak electric field. The electric field in the SRF cavity oscillates with a frequency of 1.3 GHz. At a defined phase the photocathode is struck by short light pulses (some ps length) from a frequency-doubled (at 515 nm) laser locked to the frequency of the of the SRF cavity. During emission the electron pulses are subject to rapid acceleration with gradients on the order of 20 MV/m and reach relativistic velocities after a couple of cm. The goal for the ERL accelerator is to generate a beam of ultra-short electron pulses with high average power and high brightness. After usage of the beam in radiation generation processes the electron pulses are guided back through the main acceleration section and the energy is recovered and fed to freshly generated pulses from the electron source. For this electron source, the photocathode material should exhibit high QE for visible drive laser wavelength and low intrinsic emittance to reach high brightness. To keep the emittance low, the substrate and photocathode film must be smooth. Additionally, to ensure stable operation, the photocathode must be robust to the environment within the photoinjector. To fulfill these requirements, several ultra-high vacuum (UHV) systems have been built to grow, analyse and transfer Cs-K-Sb photocathodes into the accelerator. The interconnection between all these steps requires characterisation techniques that are compatible with the accelerator requirements. For example, destructive methods cannot be applied, meaning that the range of available tools is limited. Nevertheless, the alkali co-deposition procedure developed at HZB and the adopted characterisation techniques have demonstrated the possibility to obtain reproducible photocathodes with high QE^8^. In spite of these notable achievements, severe issues remain regarding the production of single-crystalline samples with a controlled amount of defects and impurities^9–12^. The growth procedures that are routinely adopted nowadays^6,8,9^ do not allow for an *a priori* definition of the obtained stoichiometry and crystal structure starting from a given composition. As a result, in place of the desired single crystals, surface-disordered and polycrystalline photocathodes are often grown, which inhibit reproducible characterisation and in-depth understanding of the intrinsic material properties, which determine the operational characteristics of photocathodes.

*Ab initio* electronic-structure theory is ideally suited to fill this gap. In a recent work on CsK_2_Sb we demonstrated that all-electron density-functional theory (DFT) and many-body perturbation theory (MBPT) are able to unravel the electronic structure and the excitations of multi-alkali antimonides with unprecedented insight^13^. Most importantly, these methods are truly predictive, as they do not require the *a priori* knowledge of any empirical parameter of the material but only its chemical composition and crystal structure. In this context, single-crystalline solids represented by unit cells including a few atoms and satisfying a large number of symmetry operations are most efficiently treated. These structures, however, hardly match realistic multi-alkali antimonide photocathodes, where different crystal structures and stoichiometries often coexist and where the quality of the obtained materials is largely limited by the experimental conditions. The massive presence of defects in the samples is a typical characteristic of the grown photocathodes which is hardly predictable *a priori* and represents therefore a major complication from a theoretical perspective. Also experimentally, the strict conditions for *in situ* growth, characterisation, and operation requested by the samples limit the possibilities for an accurate determination of their stoichiometry and composition even through well-established techniques such as x-ray photoelectron spectroscopy (XPS)^14–16^. Overcoming this issue represents a challenge for the near future that demands new viable strategies to gain insight into the fundamental characteristics of photocathode materials, in spite of the limited knowledge of their structure and stoichiometry. First-principles methods for electronic structure theory can play a prominent role in this context, owing to their unprecedented predictive power in comparison with any other theoretical approach. For this reason, substantial advances can be achieved, by searching, identifying, and unravelling correlations between the crystalline multi-alkali antimonides modelled *ab initio* and experimentally grown photocathodes. In this way it is possible to create a virtuous circle between theory and experiments where the former does not simply fit the results of the latter but is able to provide on its own information about the materials properties. In return, data generated in the lab can be compared to the results of calculations to rationalise the properties of the photocathode in realistic growth and operational conditions.

Following this strategy, here we present a first step in this direction. In *ab initio* calculations based on DFT and MBPT, we focus on the ideal face-centred-cubic (FCC) phases of Cs_3_Sb, Cs_2_KSb, and CsK_2_Sb, and investigate their electronic structure in the valence and core region. Experimentally, QE measurements coupled with XPS are used to determine correlations between the growth procedure, the material properties, and the photocathode characteristics, including their composition, stoichiometry, and oxydation state^8,17,18^. The analysis of core states enabled by the adopted all-electron DFT formalism provides all the ingredients for a qualitative comparison with the collected XPS data on the grown photocathodes in view of new, cost efficient strategies to predict and characterise advanced electron source materials.

## Results

### Structural properties

**Figure 1:**
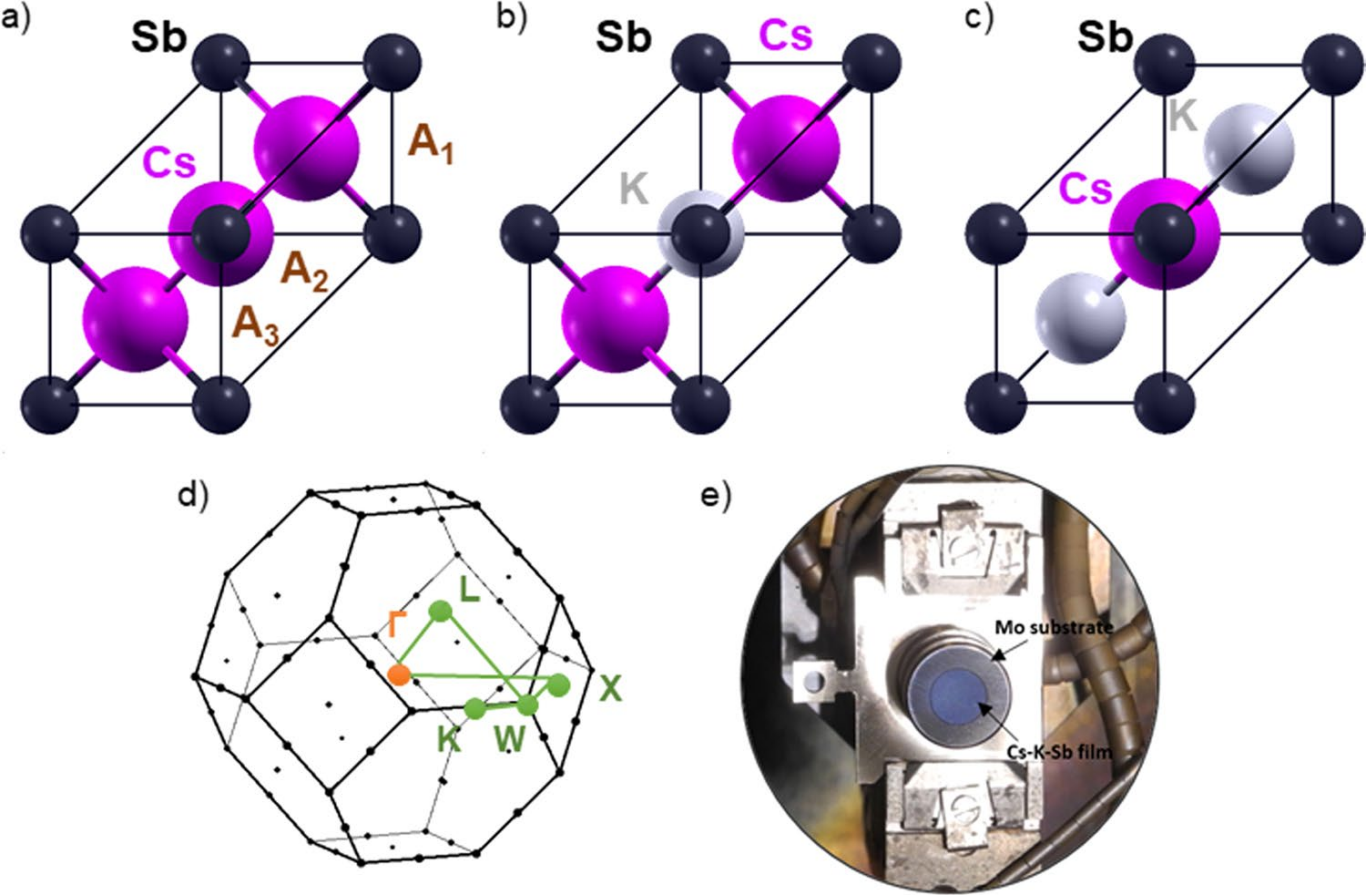
Ball-and-stick representation of primitive FCC unit cells of (**a**) Cs_3_Sb, (**b**) Cs_2_KSb, and (**c**) CsK_2_Sb. Sb atoms, depicted in black, are the origin of the cell at Wyckoff position (0,0,0), while Cs and K atoms (magenta and grey spheres, respectively) are located at crystal coordinates (1/2, 1/2, 1/2) and ±(1/4, 1/4, 1/4), labelled A_1_, A_2_, and A_3_ in panel (a). (**d**) Brillouin zone of the adopted FCC unit cell with the high-symmetry points and the path connecting them marked in colour. (**c**) Image of polycrystalline Cs-K-Sb photocathode film deposited on a Mo plug substrate, and mounted on a flag style sample holder.

Caesium-potassium-antimonides are known to crystallise in a cubic crystal struture with 16 atoms in the unit cell^19–21^. The structure of Cs_3_Sb first resolved in the pioneering study by Jack and Wachtel^19^ is characterised by 8 sites occupied by an equal number of Cs atoms and by additional 8 sites with random Cs and Sb occupations. In our first-principles calculations we consider an idealised FCC crystal structure for stiochiometric Cs_3_Sb, Cs_2_KSb, and CsK_2_Sb (see Fig. 1a–c) also adopted in previous theoretical studies^13,22^. In this FCC lattice, the Sb atoms are located at the origin of the unit cell with Wycoff coordinates (0,0,0), while the alkali species are found at (1/2, 1/2, 1/2) and ±(1/4, 1/4, 1/4). We denote these sites as A_2_, A_1_, and A_3_, respectively, as shown in Fig. 1a. From a crystallographic viewpoint, A_1_ and A_3_ represent equivalent sites. In Cs_3_Sb, where all these positions are occupied by Cs atoms, there are two inequivalent Cs atoms in the unit cell. We consider Cs_2_KSb and CsK_2_Sb structures such that the two alkali species occupy inequivalent sites, as visualised in Fig. 1b,c.

The volume optimisation carried out from DFT for the three alkali antimonide structures shown in Fig. 1a–c shows a clear trend between the lattice parameter *a* and the relative amount of Cs and K atoms. The largest lattice parameter is found in Cs_3_Sb, where *a* = 9.38 Å, and its value monotonically decreases in Cs_2_KSb (*a* = 9.23 Å) and in CsK_2_Sb (*a* = 8.76 Å), in agreement with x-ray diffraction measurements^23^. The correlation between lattice parameter and composition of caesium-potassium-antimonides was pointed out by McCarrol in 1961^21^: According to chemical intuition, the larger atomic radius of Cs compared to K promotes an increase of the unit cell volume. Our DFT results qualitatively match the trend reported in that first experimental study^21^, and, in agreement with previous DFT results^24^ performed on the cubic cell proposed by Jack and Wachtel^19^, validate our choice of the unit cell. Also the antimony-alkali bond lengths reproduce this behaviour. The distance between Sb and the alkali atom occupying the site A_1_ and equivalently A_3_ (see Fig. 1a), decreases monotonically with the relative amount of Cs atoms in the unit cell. In Cs_3_Sb the Sb-Cs distance is 4.06 Å, while in Cs_2_KSb and in CsK_2_Sb the bond length between Sb and Cs is equal to 4.00 Å and 3.79 Å, respectively.

### Electronic structure

**Figure 2:**
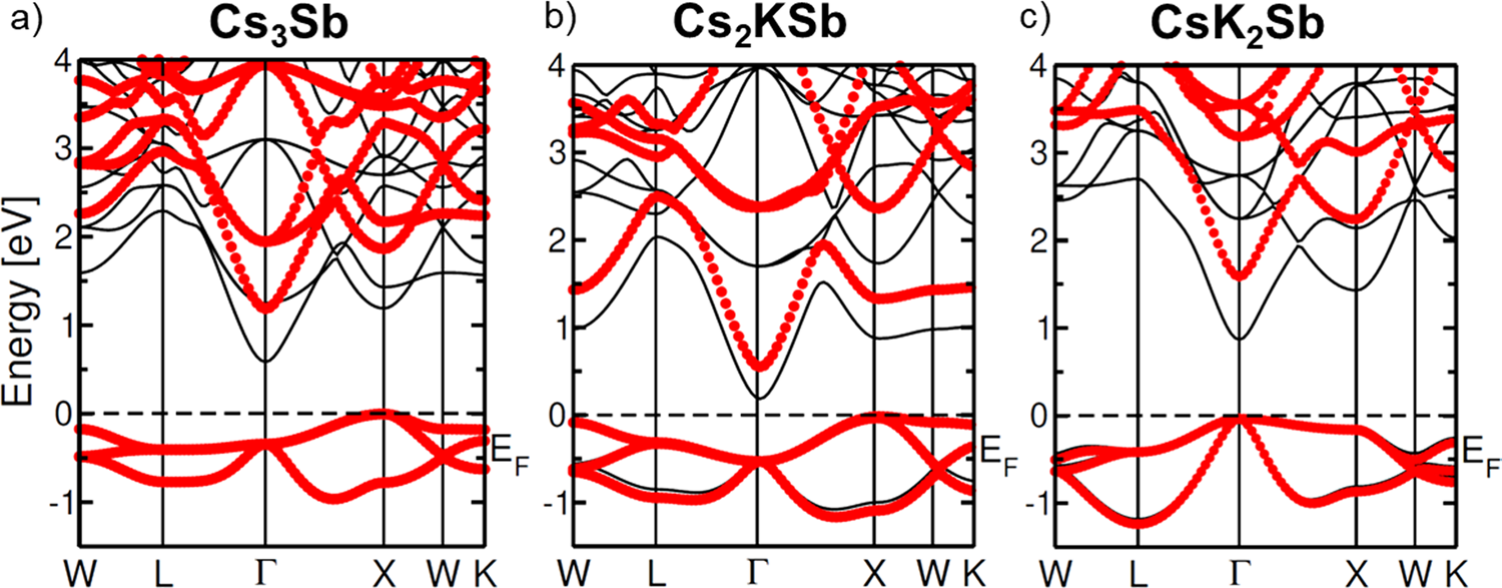
Band structures of Cs_3_Sb (**a**), Cs_2_KSb (**b**), and CsK_2_Sb (**c**). Results from DFT calculations are indicated by black lines while band structures including the quasi-particle correction as computed from *G*_0_*W*_0_ are represented by red circles. The Fermi energy (*E*_*F*_) is set to zero at the VBM.

The electronic band structures of the Cs-based materials considered in this work, as calculated from DFT (black lines) and from *G*_0_*W*_0_ (red dots), are shown in Fig. 2. The high-symmetry points reported in abscissa and the path connecting them are highlighted in the Brillouin zone depicted in Fig. 1d. We notice that qualitatively the results obtained from DFT and *G*_0_*W*_0_ are almost identical. However, quantitatively, the quasi-particle (QP) gaps obtained from *GW* are in all cases about twice as large as the DFT ones, as summarised in Table 1. CsK_2_Sb has the largest QP gap of 1.62 eV (0.92 eV from DFT, see also ref. ^13^) followed by Cs_3_Sb with a band gap 1.18 eV (0.59 eV from DFT) and finally by Cs_2_KSb with a QP gap of 0.57 eV (0.18 eV from DFT). Among these three materials, only CsK_2_Sb is characterised by a direct band gap at $\Gamma$, in agreement with earlier DFT results^24^. The other two systems have an indirect band gap between X and $\Gamma$, where the valence-band maximum (VBM) and the conduction-band minimum (CBm) appear, respectively. This finding suggests that reducing the relative amount of K leads to a shift of the VBM from the zone center $\Gamma$ to the zone edge X. This feature has an influence of the optical properties of the materials as well, as seen by the optical gaps reported in Table 1. These values, as computed from *G*_0_*W*_0_ range from 1.08 eV in Cs_2_KSb to 1.62 eV in CsK_2_Sb, where the optical and fundamental gaps coincide. Interestingly, the optical gap of Cs_3_Sb, being 1.53 eV, is only less than 100 meV smaller compared to CsK_2_Sb. Focusing more closely on the band structures, we notice that the dispersion of the valence bands increases significantly going from Cs_3_Sb, where the valence is spread over an energy range of about 1 eV, to CsK_2_Sb, where the highest-occupied bands extend over a region of almost 1.3 eV. While in the former case, and analogously also in Cs_2_KSb, the largest band dispersion occurs along the path connecting $\Gamma$, X, and W. In the band structure of CsK_2_Sb (Fig. 2c) one of the valence bands has a pronounced minimum around L and an almost parabolic shape in the vicinity of this point, along the path connecting W to $\Gamma$. In the conduction region we notice that the lowest unoccupied band in Cs_2_KSb does not cross the next one at higher energy, contrary to both Cs_3_Sb and CsK_2_Sb where this intersection is visible along the $\Gamma$-*X* path (see Fig. 2).

**Table 1:** Character and values of the fundamental gap (*E*_*gap*_) and optical gap (*E*_*opt*_) obtained from DFT and *G*_0_*W*_0_.

	*E*_*gap*_ character	${{\boldsymbol{E}}}_{{\boldsymbol{gap}}}^{{\boldsymbol{DFT}}}$	${{\boldsymbol{E}}}_{{\boldsymbol{gap}}}^{{{\boldsymbol{G}}}_{{\bf{0}}}{{\boldsymbol{W}}}_{{\bf{0}}}}$	${{\boldsymbol{E}}}_{{\boldsymbol{opt}}}^{{\boldsymbol{DFT}}}$	${{\boldsymbol{E}}}_{{\boldsymbol{opt}}}^{{{\boldsymbol{G}}}_{{\bf{0}}}{{\boldsymbol{W}}}_{{\bf{0}}}}$
Cs_3_Sb	indirect (X-$\Gamma$)	0.59	1.18	0.94	1.53
Cs_2_KSb	indirect (X-$\Gamma$)	0.18	0.57	0.67	1.08
CsK_2_Sb	direct ($\Gamma$-$\Gamma$)	0.92	1.62	0.92	1.62

In the direct-band-gap material CsK_2_Sb *E*_*gap*_ and *E*_*opt*_ obviously coincide. Energies are expressed in eV.

**Figure 3:**
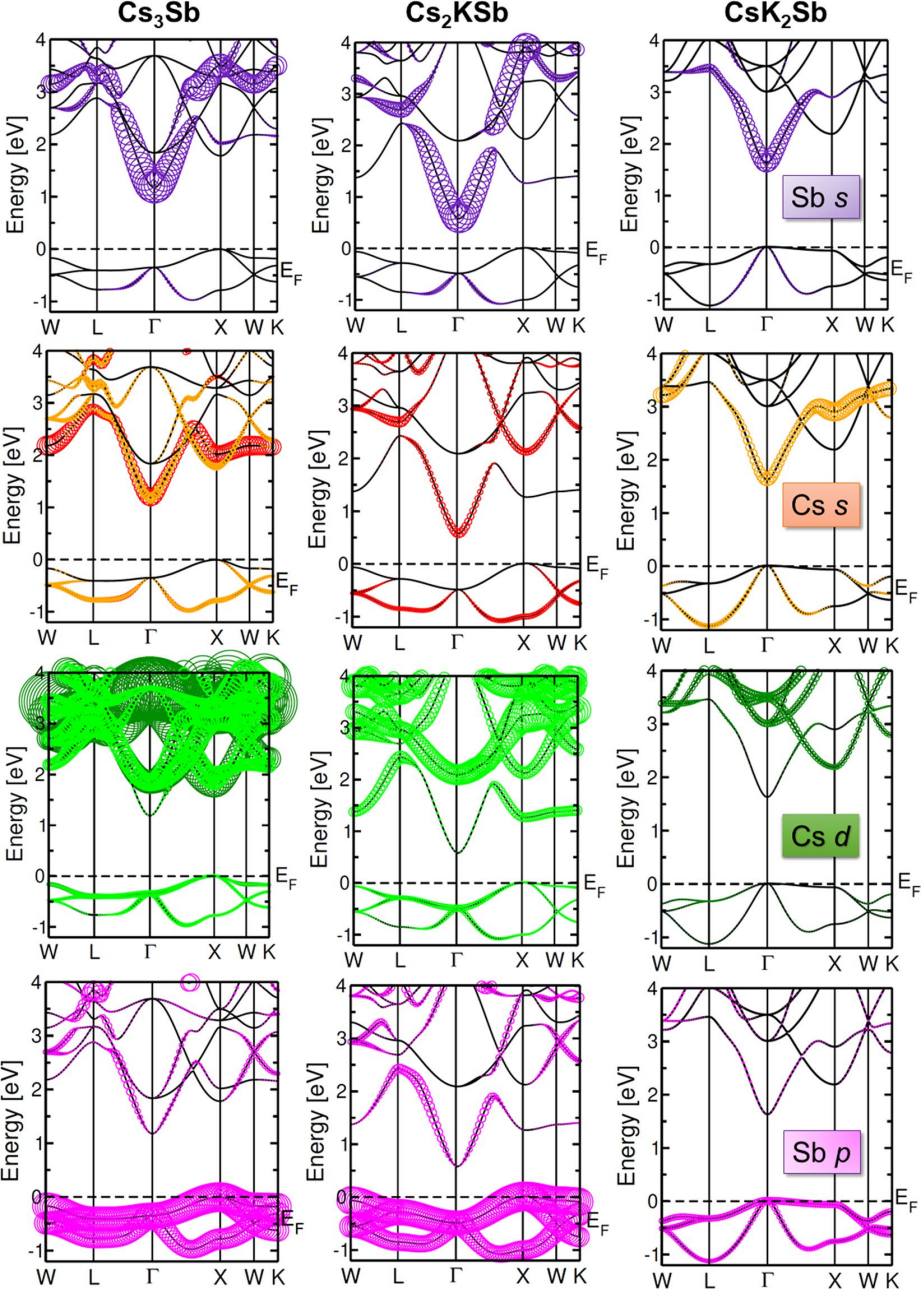
Dominant atomic character in the band structures of Cs_3_Sb (left), Cs_2_KSb (centre), and CsK_2_Sb (right). The size of the circles indicates the magnitude of the contribution to each depicted band. *s*-states from inequivalent Cs atoms in sites *A*_1_ and *A*_2_ are depicted in red and orange, respectively. *d*-states from inequivalent Cs atoms in sites *A*_1_ and *A*_2_ are depicted in dark and light green, respectively. The Fermi energy (*E*_*F*_) is set to zero at the VBM.

The electronic properties of these three materials and their relation with the Cs-K ratio can be further analysed by inspecting the band character. For this purpose, in Fig. 3 we report the most relevant atom-projected contributions to the bands of each material. While in all systems the valence bands are dominated by the partially filled Sb 4*p* shell (see also refs. ^13,22,24^), the conduction bands carry the signatures of each compound. An overview on Fig. 3 reveals that the bottom of the conduction region is dominated by the *s*-states of the Sb atoms and, to lesser extend, also of the Cs atoms. This is not surprising, considering the parabolic shape of the lowest unoccupied band in the vicinity of its minimum, which falls at the $\Gamma$ point in each considered material. The electrons in the empty Sb 5*s* shell form these bands. Also electrons from the half-filled Cs 6*s* shell contribute to the conduction bands. However, different from the Sb *s* electrons, the bands with Cs *s*-character appear more delocalised throughout the BZ. In Fig. 3 different colors are used to mark the contributions from inequivalent Cs atoms in Cs_3_Sb: Those from *A*_1_ and *A*_3_ (*A*_2_) sites are displayed in red (orange). We notice that contributions from Cs *s*-states from atoms in *A*_1_ positions appear mainly in the lowest unoccupied band. On the other hand, Cs *s*-states from atoms in *A*_2_ sites contribute not only to the bottom of the conduction but also to higher unoccupied bands. In the other two compounds, Cs_2_KSb and CsK_2_Sb, only one inequivalent Cs atoms is present. In both cases the Cs *s*-states contribute to the lowest-unoccupied band in the vicinity of $\Gamma$ being hybridised with the Sb *s*-states. Additional contributions from Cs *s*-states in these materials are provided also to higher-energy unoccupied bands, especially around the the high-symmetry points X (both), L (Cs_2_KSb), and W (CsK_2_Sb). It should also be noted that in these systems the contributions from K atoms enter into play in the conduction region^13,24^. In the bottom panels of Fig. 3 the bands with Cs *d*-character are displayed. Also in this case we adopt different colors to identify the contributions from inequivalent Cs atoms (light green for those on *A*_2_ sites and dark green for those on *A*_1_ and *A*_3_ sites). We emphasise that the weights of each state, quantified by the size of the colored circles, are represented with the same scaling ratio for each atomic character and in each material. Hence, the relative comparison between the plots displayed in Fig. 3 is quantitative. Interestingly, in the materials containing potassium, the contribution of the Cs *d*-states to the conduction bands is complementary to those of the Cs and Sb *s*-states. The Sb *d*-shell does not significantly contribute to the first few eV above the CBm, as discussed in ref. ^13^ in the case of CsK_2_Sb.

### Core spectroscopy

The adopted theoretical formalism, based on all-electron DFT^25^, allows to access the energies of all the electrons in the core. Although core energies are systematically underestimated by 10–20% in DFT^26^, relative core shifts referred to the same levels in different materials can be correlated with XPS data in a meaningful way. The calculated core level energies of each state in all the investigated materials are reported in Table 2. We separate line by line the binding energies of states with different principal and angular quantum numbers *n* and *l*. Contributions from crystallographically inequivalent Cs atoms are identified by their site in the unit cell, according to the scheme in Fig. 1a. The energies of Cs atoms on sites *A*_3_, which are equivalent to those on sites *A*_1_, are omitted. A colour code is adopted in Table 2 to visualise the magnitude of core-level shifts with respect to Cs_3_Sb. In this way it is possible to catch at a glance the rather regular patterns that do not vary depending on the quantum numbers of core states but appear instead as a general property of atoms at specific sites. The relative shift of core levels of Cs atoms on site *A*_1_ in Cs_2_KSb compared to those in Cs_3_Sb amounts to 0.27 eV. The corresponding values in Table 2 are marked in orange. The binding energies of the core states of Cs atoms on site *A*_2_ in CsK_2_Sb are shifted by −0.82 eV with respect to Cs_3_Sb and are highlighted in green in Table 2. We recall that larger binding energies correspond to deeper levels. Binding energies of core levels associated to crystallographically inequivalent Cs atoms in Cs_3_Sb are always separated in energy by 0.87 eV, with states relative to atoms on site *A*_2_ being always energetically deeper. The core shifts associated to K and Sb atoms are also insensitive to the specific core state but appear once again as a general property of the atomic species in the specific compounds. The core binding energies of antimony atoms in Cs_2_KSb and CsK_2_Sb increase with respect to those in Cs_3_Sb by different amounts of energies. In CsK_2_Sb, Sb binding energies are slightly larger by less than 0.1 eV compared to those in Cs_3_Sb (yellow boxes in Table 2. On the other hand, the shifts between Sb core states in Cs_3_Sb and Cs_2_KSb is of the order of 0.5 eV (red boxes in Table 2). The most remarkable shifts in the binding energies of core levels appear between 1*s* and 2*p* states of potassium in the two considered bi-alkali antimonides. A close inspection of the blue boxes in Table 2 reveals shifts larger than 2 eV, which are again almost constant regardless of the quantum numbers of core states. K states in Cs_2_KSb are deeper than those in CsK_2_Sb.

**Table 2:**
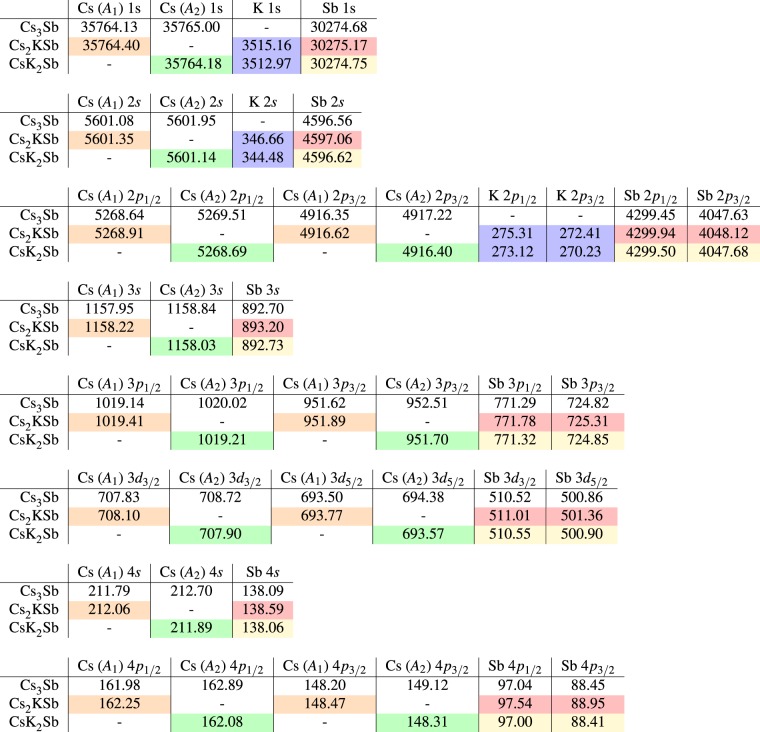
Core-level binding energies computed from DFT for all the atomic species in Cs_3_Sb, Cs_2_KSb, and CsK_2_Sb.

Inequivalent Cs atoms are identified based on their crystallographic sites (see Fig. 1). A colour code is adopted to identify patterns in the magnitude of relative shifts with respect to Cs_3_Sb: yellow denotes shifts of less than 0.1 eV, orange of 0.27 eV, red of about 0.5 eV, green of 0.8 eV, and blue by over 2 eV. All energies are expressed in eV.

**Figure 4:**
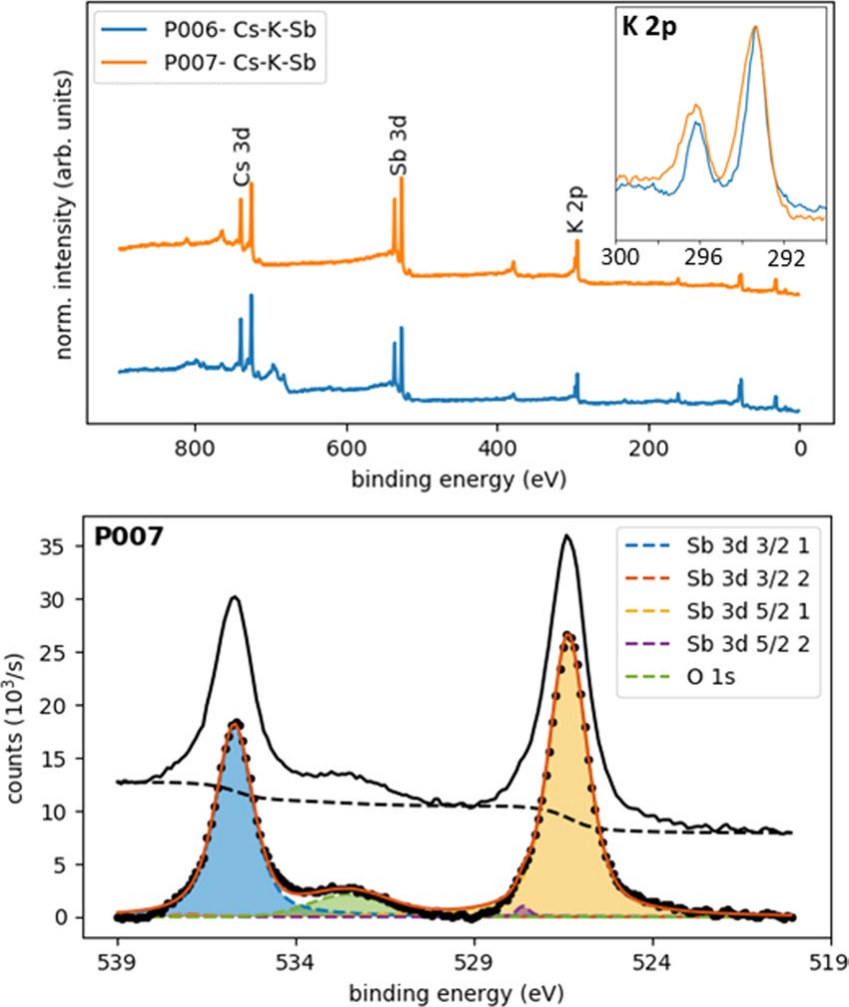
(top) XPS survey spectra of the Cs-K-Sb photocathodes P006 and P007. The photoemission lines for Cs 3*d*, K 2*p,* and Sb 3*d*, which are most relevant in the chemical analysis of the samples, are highlighted. Region scan of the K 2*p* peaks for P006 and P007 (top-right) from which peak positions, shifts and fittings are obtained. (bottom) Sb 3*d* and O 1*s* region spectrum deconvoluted by fitting peaks for O 1*s* (dashed green line) and the two antimony species: (1) Sb^3−^ and (2) Sb^(0)^. The measured signal with Shirley background are shown as a black line and a dashed black line respectively.

**Figure 5:**
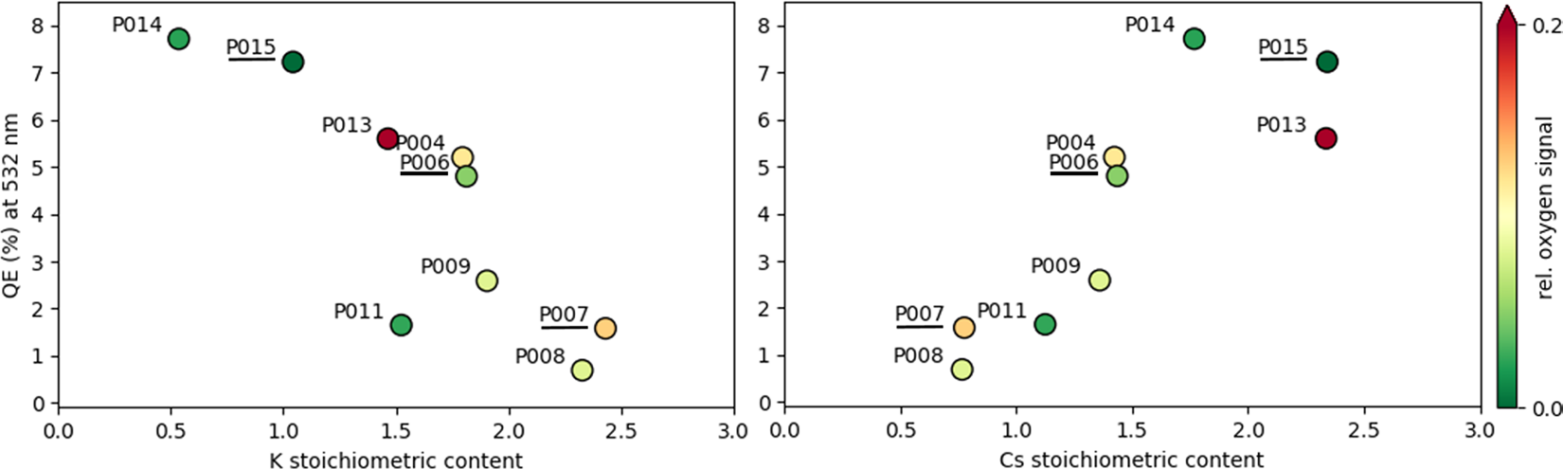
Correlation between QE, recorded under illumination with 532 nm wavelength, and stoichiometric content of (left) potassium and (right) caesium in a series of photocathode samples grown at HZB. The colour of the points, according to scale on the right, indicates the relative amount of oxygen contained in each sample.

The regular patterns observed in the computed core level shifts offer promising perspectives to monitor peak energies in XPS spectra of multi-alkali antimonide photochathodes grown for bERLinPro. Here, we consider this possibility retroactively for photocathodes P006 and P007 grown and characterised at HZB^27^. These samples are grown on a Mo substrate (see Fig. 1e) using an alkali codeposition technique recently developed for bERLinPro and described in details in ref. ^8^. The stoichiometric characterisation of the photocathodes is performed *in-situ* using XPS, which is a well established surface analysis technique that is used to determine the chemical composition of surfaces and thin films^28^. The advantages of XPS as a diagnostic technique in the context of bERLinPro are its non-destructive nature and the possibility that it gives in the employed setup to characterise the materials without exposing them to the atmosphere (more details are provided in the Experimental Section below). In Fig. 4 XPS spectra corresponding to the final stage of Cs-K-Sb growth are presented. Spectra from survey measurements of P006 and P007 (top panel) show the elemental composition of the photocathode materials. Peaks associated to specific core states are clearly visible, with those referring to Cs 3*d*, Sb 3*d*, and K 2*p* highlighted as the most prominent and identifiable features in the spectrum. The stoichiometry of the samples  is determined from the relative peak intensities of the aforementioned peaks, as reported in Table 3. More detailed region scans, such as the one shown for  K 2*p* (see inset of the top panel in Fig. 4) offer additional insight in determining peak positions and shifts, and are also useful for peak fittings. The K 2*p* peak positions in the spectra of P006 and P007 are very similar. The sizable shifts predicted by theory are actually not observed in the top panel of Fig. 4, which is not surprising, considering that photocathode samples are non-stoichiometric (see Fig. 5) and they may contain amorphous or polycrystalline regions with different stoichiometry.

**Table 3:** Chemical composition, stoichiometric content, and final QE of the Cs-K-Sb samples P006, P007, and P015 (see Fig. 5).

Sample	Sb	K	Cs	QE (%)
P006	1	1.8	1.4	4.8
P007	1	2.4	0.8	1.6
P015	1	1.0	2.3	7.2

The QE is measured at 2.33 eV.

Real photocathodes are also subject to the possibility of oxygen contamination due to residual gases of K and Cs dispensers. A detailed region spectrum of the Sb 3*d* and O 1*s* regions for P007 is shown in the bottom panel of Fig. 4. The oxygen contamination of a sample can be calculated from this plot by extracting the relative peak intensity of the O 1*s* and Sb 3*d*_5/2_ states. Since the binding energies shown in the plot in Fig. 4 (bottom panel) are so close, the spectrum has been deconvoluted to identify the individual contributions of the O 1*s* and the two Sb species (3*d*_3/2_ and 3*d*_5/2_) with oxidation state Sb^3−^ (blue and orange dashed lines) and Sb^(0)^ (red and purple dashed lines). For reference, the raw measured data is reported with offset (black solid line) together with the Shirley background (black dashed line), which has been subtracted to obtain the deconvoluted spectrum in the foreground. From this graph it is clear that the P007 sample is dominated by antimony atoms with Sb^3−^ oxidation state. Very minor contributions from Sb^(0)^ can be seen only for the 3*d*_5/2_ electron at 527.6 eV. For P007 we also see some oxygen contamination identified by the broad peak centred at 532.6 eV, which is visible also in the stoichiometric analysis reported below (see Fig. 5). The analysis of Fig. 4 is exemplary to the characterization of a photocathode sample which highlights the chemical nature of the grown material, which contain oxygen impurities and non-stoichoimetric composition, as discussed below with reference to Fig. 5.

In Fig. 5 the correlation between QE, measured under illumination with 532 nm wavelength, and stoichiometric content of Cs and K atoms is presented for a selected number of Cs-K-Sb samples grown at HZB (for details, see the Experimental Setup below). The relative oxygen contamination (ratio of O 1*s* to Sb 3*d* peak intensity) of each sample is indicated according to the colour scale on the right-hand side of the figure: Green points correspond to oxygen-free cathodes, while red dots are referred to samples with up to 0.2 relative oxygen content. For pristine single crystals, such as those modelled by DFT, corresponding to CsK_2_Sb and Cs_2_KSb, respectively, one would expect to see two distinct clusters of samples around $x=1$ and $x=2$, where *x* indicates the relative K or Cs content. In reality, the grown samples are polycrystalline in nature, thus composed of grains of the two different stoichiometries. The presence of other crystal phases rather than the most stable cubic ones considered in the DFT calculations cannot be excluded. The stoichiometric content of the samples presented in Fig. 5 is the mean value averaged over a surface of several mm^2^ ^27^. As a result, the points on both panels in Fig. 5 are rather scattered, spanning the entire region between $x=1$ and $x=2$ and also expanding above and below it. We notice a correlation between the amount of oxygen content and the QE, in particular in the samples P014 and P015, which exhibit highest purity and, concomitantly, highest efficiency among the data points reported in Fig. 5. On the other hand, the relatively large QE of P013 and, conversely, the relatively low QE of P011, do not allow us to draw general conclusions. Overall, in the presented pool of samples, those with stoichiometry close to the Cs_2_KSb exhibit the highest QE. We finally remark that while from Fig. 5 one may evince that no K content leads to a maximized QE, for the operation of the cathodes in the bERLinPro accelerator, specific constraints in term of material robustness and thermal stability have to be fulfilled. Such criteria are met by Cs-K-Sb compounds but not entirely by Cs_3_Sb^1^.

We conclude this section by benchmarking the spin-orbit splitting, which is clearly visible in the calculated values of core binding energies in Table 2, as well as in the measured XPS spectra (see Fig. 4), against tabulated references. In this way, we can use spin-orbit splitting, which is known to be an atomic property, as a parameter to validate both computed and measured values. We summarize this comparison in Table 4, where we contrast the results of spin-orbit splitting for three selected states (Cs 3*d*, K 2*p*, and Sb 3*d*) in Cs-K-Sb materials (Cs_2_KSb and CsK_2_Sb calculated compounds) with those reported in ref. ^29^ for the corresponding elemental crystals. The comparison with DFT values reveals a very good agreement for K 2*p* and Sb 3*d*, with discrepancies of less than 5%. The computed spin-orbit splitting for Cs 3*d* is instead overestimated by DFT by slightly more than 10%. Analogous trends are obtained also for the experimental values, where the discrepancy between Cs_2_KSb and CsK_2_Sb can be attributed to the uncertainty of the measurement. In this case, the splittings of K 2*p* and Sb 3*d* are smaller than the reference. It should also be noticed that the experimental data reported in Table 4 are obtained from samples P007 and P015, which are not perfectly stoichiometric (see Fig. 5). Although spin-orbit splittings are not meaningful to identify different stoichiometries, the mutual agreement between DFT and XPS results, also with respect to the tabulated values, enforces the connection between *ab initio* theory and survey experiments on the grown photocathodes, which are very specific to the application and operation in a photoinjector.

**Table 4:** Spin-orbit splitting (Δ) within Cs 3*d*, K 2*p*, and Sb 3*d* states computed from DFT and extracted from XPS measurements on CsK_2_Sb and Cs_2_KSb stiochiometries (samples P007 and P015 in Fig. 5 and Table 3).

	Δ DFT [eV]		Δ XPS [eV]		Δ Ref. ^29^ [eV] Elemental crystal
	Cs_2_KSb	CsK_2_Sb	CsK_2_Sb	Cs_2_KSb	
Cs 3*d*	14.33	14.33	13.9	14.0	12.67
K 2*p*	2.90	2.89	2.8	2.8	2.77
Sb 3*d*	9.65	9.66	9.3	9.3	9.34

Tabulated values from ref. ^29^ for elemental bulk materials are reported in the third column.

## Summary and Conclusions

The results presented in this work demonstrate the ability of *ab initio* methods for solid-state theory to unveil the fundamental physical properties of photocathode materials. By adopting state-of-the art approaches based on all-electron DFT and MBPT in the *GW* approximation, we have computed the electronic properties of three alkali antimonides materials for photocathodes, namely Cs_3_Sb, Cs_2_KSb, and CsK_2_Sb. A quantitative estimate of the band gap has revealed that all these systems are characterised by rather low quasi-particle band gaps, ranging from 0.57 eV in Cs_2_KSb to 1.62 eV in CsK_2_Sb. The character of the band gap changes depending on the relative content of potassium, being direct at $\Gamma$ in CsK_2_Sb and becoming indirect in the other two compounds. The study of the band character reveals valence bands dominated by antimony *p* states (see also ref. ^13^) and conduction bands with relevant contributions from both Cs and and Sb *s* states at lower energies, and from Cs *d* states further away from the Fermi energy. The analysis of the binding energies of the core states shows regular patterns in the shifts associated to individual atomic species in specific crystallographic sites. In particular, our DFT results indicate sizable shifts, of the order of 2 eV and 0.5 eV, for K 2*p* and Sb 3*d* states between Cs_2_KSb and CsK_2_Sb. These trends can be used to identify correlations between *ab initio* theory and measurements on bi-alkali antimonide photocathode samples. In this regard we have shown the result of XPS characterization measurements performed on a set of Cs-K-Sb photocathodes prepared for the bERLinPro project. The XPS survey spectra of the grown samples reveal non-stroichiometric compositions as well as the presence of oxygen contaminants, which in turn determine the oxidation states of the Sb species. Finally, we have shown how the stoichiometry and the chemical composition of the cathodes, including their relative oxygen content, is related to the measured QE.

In conclusion, our combined theoretical and experimental study shows that correlations between *ab initio* results and measurements of core binding energies can be drawn in order to characterise multi-alkali antimonide photocathodes. We emphasise that this connection is particularly relevant given the limited freedom for *in situ* and *in operando* characterization of photocathode materials for accelerator applications. The possibility to grow purely stoichiometric photocathodes is achievable only using X-ray beamlines equipped with suitable and yet non-transferable setups^12^, which enhances the role of *ab initio* theory in the whole optimization process. While at the present status, we have explored from first principles only ideal stoichiometric bulk materials, further studies are requested to bridge the gap with experiments. Additional calculations addressing non-stoichiometric compounds are expected to be significantly more demanding from a computational perspective but more relevant to finally bridge the gap to experiments. Novel techniques such as high-throughput screening and machine learning are expected to provide an important aid towards the achievement of this goal. In this perspective, the results of this work represent the first step towards establishing a robust connection between experimental preparation and characterisation of photocathodes, *ab initio* prediction of their electronic structure, and modeling of the emission and beam formation processes in operational conditions.

## Methods

### Theoretical background and computational details

The first-principles results presented in this work are obtained in the framework of density-functional theory (DFT) and many-body perturbation theory (MBPT). DFT is based on the Hohenberg-Kohn theorems^30^ and is implemented according to the Kohn-Sham (KS) scheme^31^, which consists of mapping the many-electron problem into a set of independent-particle equations for the electronic wave-functions of each electron *n* in the system at each **k**-point in the Brillouin zone:

1 $$[\,-\,\frac{{\nabla }^{2}}{2}+{v}_{s}({\bf{r}})]\,{\varphi }_{n{\bf{k}}}({\bf{r}})={\varepsilon }_{n{\bf{k}}}^{KS}{\varphi }_{n{\bf{k}}}({\bf{r}}),$$

where ${\varepsilon }_{n{\bf{k}}}^{KS}$ is the KS energy per particle. Eq. (1) is expressed in atomic units, which are adopted from now on. On the left-hand side of Eq. (1), in addition to the kinetic-energy operator, we find the effective potential per particle ${v}_{s}({\bf{r}})$, which consists of the sum of three terms: ${v}_{s}({\bf{r}})={v}_{ext}({\bf{r}})+{v}_{H}({\bf{r}})+{v}_{xc}({\bf{r}})$. The external potential *v*_*ext*_ (**r**) includes the interaction between the negatively-charged electrons and the positively-charged nuclei. The Hartree potential ${v}_{H}({\bf{r}})$ accounts for the (mean-field) Coulomb between the electrons, and ${v}_{xc}({\bf{r}})$ is the exchange-correlation (xc) potential. Since the exact form of ${v}_{xc}({\bf{r}})$ is unknown, this term in Eq. (1) has to be approximated. In this work, we adopt the generalised gradient approximation (GGA) as implemented in the Perdew-Burke-Ernzerhof parameterisation^32^. A relevant aspect in the solution of Eq. (1) is the choice of the basis set. Here, we adopt the linearized augmented planewave plus local-orbital (LAPW + lo) method, which allows for an explicit treatment of core electrons. Their wave-functions are described by solution of the Dirac equation in the spherically symmetric potential given by the nuclei. Further details are provided in refs. ^25,33^.

The *GW* approximation^34^ in the single-shot perturbative approach *G*_0_*W*_0_^35^ is adopted to estimate the quasi-particle correction to the valence and conduction states in the gap region. In this formalism, the quasi-particle energies of each electronic band ${\varepsilon }_{i{\bf{k}}}^{QP}$ are computed from the electronic self-energy $\Sigma$ as:

2 $${\varepsilon }_{i{\bf{k}}}^{QP}={\varepsilon }_{i{\bf{k}}}^{KS}+{Z}_{i{\bf{k}}}[\Re {\Sigma }_{i{\bf{k}}}({\varepsilon }_{i{\bf{k}}}^{KS})-{V}_{i{\bf{k}}}^{xc}],$$

where *Z*_*i***k**_ is the renormalization factor accounting for the energy-dependence of the self-energy and ${\varepsilon }_{i{\bf{k}}}^{KS}$ are the solutions of the Kohn-Sham equations for the given states. For the derivation of Eq. (2) and additional information we refer for review to refs. ^36,37^. The details of the implementation of *GW* in an all-electron formalism are reported in refs. ^38,39^ Binding energies of core states are computed from DFT as the the energy of each level with opposite sign. No QP correction is applied in this case, as the extremely localised character of core electrons requires a many-body treatment that goes beyond the *GW* approximation^40^. Absolute values of core binding energies computed from DFT are therefore systematically underestimated by 10–20% compared to experiments.

All calculations presented in this work are performed with the exciting code^25^. The muffin-tin (MT) radii of all the atomic species involved (Cs, K, and Sb) are set to 1.65 bohr and a plane-wave basis-set cutoff ${R}_{{\rm{MT}}}{G}_{{\rm{\max }}}=8.0$ is employed. For ground-state calculations the Brillouin zone (BZ) is sampled using a homogeneous cubic **k**-mesh with 8 points in each direction, corresponding to overall 29 points considering the symmetry operations. The optimised volume of each unit cell is obtained by fitting DFT results obtained at varying lattice parameters with the Birch-Murnaghan equation of state^41,42^. To calculate the quasi-particle band-structure within the *G*_0_*W*_0_ approximation, a 4 × 4 × 4 **k**-mesh is adopted without exploiting symmetries, for a total of 64 **k**-points. Unit cells and BZs are visualised with the XCrysDen software^43^.

### Experimenatal setup

The experimental results presented in this work come from the Photocathode Preparation and Analysis system at HZB. The system is composed of two main chambers. The preparation chamber houses the evaporation sources: an effusion cell to evaporate high purity Sb beads and SAES dispenser sources for alkali deposition. A spectral response setup is also attached to the preparation chamber to measure the QE(*λ*), ranging from 1.77 eV (700 nm) to 3.35 eV (370 nm). For a full description of the setup, see refs. ^27,44^. The setup for QE measurements is composed of the following: a tunable white light source and monochromator for stimulating photoemission, a pickup anode and picoammeter (Keithley model 6487) to measure the extracted photocurrent, and a calibrated photodiode power meter (Thor-Labs PM100D and S130VC) to measure the power of the light. At the end of the photocathode growth procedure a photocurrent measurement is taken. Green light (2.33 eV) is irradiated onto the sample and a bias voltage is applied between the earthed photocathode and the pick-up anode; the current is then measured by the picoammeter. A dark current measurement is taken and subsequently subtracted from the current measurement to obtain the true photocurrent, from which the QE is calculated. In this way, the QE values presented in this paper are obtained.

The analysis chamber houses an electron analyser (Specs Phoibos 100 2D-CCD) and x-ray source (Specs XR 50) for XPS measurements. The chamber is directly attached to the preparation chamber thus allowing for *in-situ* growth and analysis. A full description of the setup is available at ref. ^27^. In this work the samples have been excited by non-monochromatic Al K*α* radiation. The survey spectra were obtained with a constant pass energy of 20 eV, and detailed region spectra with 10 eV. The uncertainty that arises from the corresponding spectra are 0.37 eV and 0.19 eV respectively.

In an XPS experiment, a sample is irradiated with X-ray photons of known energy, and the resulting photoelectrons liberated by this process are collected and their kinetic energy is recorded with an electron spectrometer. A spectrum is generated of electron counts per second as a function of kinetic energy and then, using Eq. (3), converted into binding energy (see Fig. 4).

3 $${}_{KE}=h\nu -{E}_{BE}-e\phi ,$$

where *E*_*KE*_ is the kinetic energy of the emitted electron, $h\nu$ is the excitation photon energy, *E*_*BE*_ is the binding energy of the core state with respect to the Fermi level and $\phi$ is the work function of the spectrometer.

The binding energy is dependent on the element from which the electron was emitted, and therefore the spectra provides information of the electronic structure of the sample material from which elemental and chemical composition and can be determined^45^. To obtain the stoichiometric content for the photocathodes presented in this work, the relative peak intensity of the Sb 3*d*, K 2*p* and Cs 3*d* peaks from the XPS survey spectra were quantitatively analysed using CasaXPS in conjunction with a custom PYTHON code^27^. The ratio for two concentrations (*c*_*a*_ and *c*_*b*_) were determined using Eq. (4):

4 $$\frac{{c}_{a}}{{c}_{b}}=\frac{{I}_{a}}{{I}_{b}}\frac{{\sigma }_{ax,B}{\lambda }_{IMFP,B}}{{\sigma }_{ax,A}{\lambda }_{IMFP,A}},$$

where *I*_*A*_ and *I*_*B*_ are the intensity of two atomic species Cs and K, *σ*_*ax*_ is the excitation cross-section, *λ*_*IMFP*_ is the inelastic mean free path.

In this work, cross-sections (*σ*_*ax*_) from Scofield are used and IMFPs (*λ*_*IMFP*_) are obtained from the program SESSA using the TPP-2M formula^46,47^. A custom PYTHON code was used to first carry out a normalisation of the data using the analyser transmission function and then a standardised Shirley background subtraction was performed, followed by an integration of the peak intensity *I*. In this way, the stoichiometric content of Cs and K were calculated for the photocathodes presented in this work.

## Data Availability

Data are available upon reasonable requests to the corresponding authors.
